# Discovery of biomarkers for glycaemic deterioration before and after the onset of type 2 diabetes: descriptive characteristics of the epidemiological studies within the IMI DIRECT Consortium

**DOI:** 10.1007/s00125-019-4906-1

**Published:** 2019-06-15

**Authors:** Robert W. Koivula, Ian M. Forgie, Azra Kurbasic, Ana Viñuela, Alison Heggie, Giuseppe N. Giordano, Tue H. Hansen, Michelle Hudson, Anitra D. M. Koopman, Femke Rutters, Maritta Siloaho, Kristine H. Allin, Søren Brage, Caroline A. Brorsson, Adem Y. Dawed, Federico De Masi, Christopher J. Groves, Tarja Kokkola, Anubha Mahajan, Mandy H. Perry, Simone P. Rauh, Martin Ridderstråle, Harriet J. A. Teare, E. Louise Thomas, Andrea Tura, Henrik Vestergaard, Tom White, Jerzy Adamski, Jimmy D. Bell, Joline W. Beulens, Søren Brunak, Emmanouil T. Dermitzakis, Philippe Froguel, Gary Frost, Ramneek Gupta, Torben Hansen, Andrew Hattersley, Bernd Jablonka, Jane Kaye, Markku Laakso, Timothy J. McDonald, Oluf Pedersen, Jochen M. Schwenk, Imre Pavo, Andrea Mari, Mark I. McCarthy, Hartmut Ruetten, Mark Walker, Ewan Pearson, Paul W. Franks, for the IMI DIRECT Consortium

**Affiliations:** 1 Department of Clinical Sciences, Lund University Diabetes Centre, Genetic and Molecular Epidemiology Unit, CRC, Skåne University Hospital Malmö, Building 91, Level 10, Jan Waldenströms gata 35, SE-205 02 Malmö, Sweden; 2 Oxford Centre for Diabetes, Endocrinology and Metabolism, Radcliffe Department of Medicine, University of Oxford, Oxford, UK; 3 Population Health & Genomics, Medical Research Institute, University of Dundee, Dundee, DD1 9SY UK; 4 Department of Genetic Medicine and Development, University of Geneva Medical School, Geneva, Switzerland; 5 Institute of Genetics and Genomics in Geneva (iGE3), University of Geneva, Geneva, Switzerland; 6 Swiss Institute of Bioinformatics, Geneva, Switzerland; 7 Institute of Cellular Medicine (Diabetes), Newcastle University, Newcastle upon Tyne, UK; 8 The Novo Nordisk Foundation Center for Basic Metabolic Research, Faculty of Health and Medical Science, University of Copenhagen, Copenhagen, Denmark; 9 NIHR Exeter Clinical Research Facility, University of Exeter Medical School, Exeter, UK; 10 Department of Epidemiology and Biostatistics, Amsterdam Public Health Research Institute, VU University Medical Center, Amsterdam, the Netherlands; 11 Department of Medicine, University of Eastern Finland and Kuopio University Hospital, Kuopio, Finland; 12 Department of Clinical Epidemiology, Bispebjerg and Frederiksberg Hospital, the Capital Region, Copenhagen, Denmark; 13 MRC Epidemiology Unit, University of Cambridge School of Clinical Medicine, Cambridge, UK; 14 Faculty of Health Sciences, University of Southern Denmark, Odense, Denmark; 15 Department of Bio and Health Informatics, Technical University of Denmark, Lyngby, Denmark; 16 Wellcome Centre for Human Genetics, University of Oxford, Oxford, UK; 17 Department of Clinical Sciences, Clinical Obesity, Skåne University Hospital Malmö, Lund University, Malmö, Sweden; 18 Novo Nordisk A/S, Søborg, Denmark; 19 HeLEX, Nuffield Department of Population Health, University of Oxford, Old Road Campus, Headington, Oxford, UK; 20 Research Centre for Optimal Health, Department of Life Sciences, University of Westminster, London, UK; 21 Institute of Neurosciences, National Research Council, Padova, Italy; 22 Institute of Epidemiology II, Helmholtz Zentrum Muenchen, German Research Center for Environmental Health (GmbH), Neuherberg, Germany; 23 The Novo Nordisk Foundation Center for Protein Research, University of Copenhagen, Copenhagen, Denmark; 24 Department of Genomics of Common Disease, School of Public Health, Imperial College London, London, UK; 25 CNRS, Pasteur Institute of Lille, University of Lille, Lille, France; 26 Nutrition and Dietetics Research Group, Department of Medicine, Division of Diabetes, Endocrinology and Metabolism, Imperial College London, Hammersmith Campus, London, UK; 27 Institute of Biomedical and Clinical Science, University of Exeter Medical School, Exeter, UK; 28 Sanofi-Aventis Deutschland GmbH, R&D, Frankfurt am Main, Germany; 29 Science for Life Laboratory, School of Engineering Sciences in Chemistry, Biotechnology and Health, KTH - Royal Institute of Technology, Stockholm, Sweden; 30 Eli Lilly Regional Operations GmbH, Vienna, Austria; 31 NIHR Oxford Biomedical Research Centre, Churchill Hospital, Oxford, UK; 32 Department of Nutrition, Harvard School of Public Health, Boston, MA USA; 33 Department of Public Health & Clinical Medicine, Section for Medicine, Umeå University, Umeå, Sweden

**Keywords:** Diet, Ectopic fat, Genome, Glycaemic control, Insulin secretion, Insulin sensitivity, Personalised medicine, Physical activity, Prediabetes, Type 2 diabetes

## Abstract

**Aims/hypothesis:**

Here, we describe the characteristics of the Innovative Medicines Initiative (IMI) Diabetes Research on Patient Stratification (DIRECT) epidemiological cohorts at baseline and follow-up examinations (18, 36 and 48 months of follow-up).

**Methods:**

From a sampling frame of 24,682 adults of European ancestry enrolled in population-based cohorts across Europe, participants at varying risk of glycaemic deterioration were identified using a risk prediction algorithm (based on age, BMI, waist circumference, use of antihypertensive medication, smoking status and parental history of type 2 diabetes) and enrolled into a prospective cohort study (*n* = 2127) (cohort 1, prediabetes risk). We also recruited people from clinical registries with type 2 diabetes diagnosed 6–24 months previously (*n* = 789) into a second cohort study (cohort 2, diabetes). Follow-up examinations took place at ~18 months (both cohorts) and at ~48 months (cohort 1) or ~36 months (cohort 2) after baseline examinations. The cohorts were studied in parallel using matched protocols across seven clinical centres in northern Europe.

**Results:**

Using ADA 2011 glycaemic categories, 33% (*n* = 693) of cohort 1 (prediabetes risk) had normal glucose regulation and 67% (*n* = 1419) had impaired glucose regulation. Seventy-six per cent of participants in cohort 1 was male. Cohort 1 participants had the following characteristics (mean ± SD) at baseline: age 62 (6.2) years; BMI 27.9 (4.0) kg/m^2^; fasting glucose 5.7 (0.6) mmol/l; 2 h glucose 5.9 (1.6) mmol/l. At the final follow-up examination the participants’ clinical characteristics were as follows: fasting glucose 6.0 (0.6) mmol/l; 2 h OGTT glucose 6.5 (2.0) mmol/l. In cohort 2 (diabetes), 66% (*n* = 517) were treated by lifestyle modification and 34% (*n* = 272) were treated with metformin plus lifestyle modification at enrolment. Fifty-eight per cent of participants in cohort 2 was male. Cohort 2 participants had the following characteristics at baseline: age 62 (8.1) years; BMI 30.5 (5.0) kg/m^2^; fasting glucose 7.2 (1.4) mmol/l; 2 h glucose 8.6 (2.8) mmol/l. At the final follow-up examination, the participants’ clinical characteristics were as follows: fasting glucose 7.9 (2.0) mmol/l; 2 h mixed-meal tolerance test glucose 9.9 (3.4) mmol/l.

**Conclusions/interpretation:**

The IMI DIRECT cohorts are intensely characterised, with a wide-variety of metabolically relevant measures assessed prospectively. We anticipate that the cohorts, made available through managed access, will provide a powerful resource for biomarker discovery, multivariate aetiological analyses and reclassification of patients for the prevention and treatment of type 2 diabetes.

**Electronic supplementary material:**

The online version of this article (10.1007/s00125-019-4906-1) contains peer-reviewed but unedited supplementary material, which is available to authorised users.

## Introduction

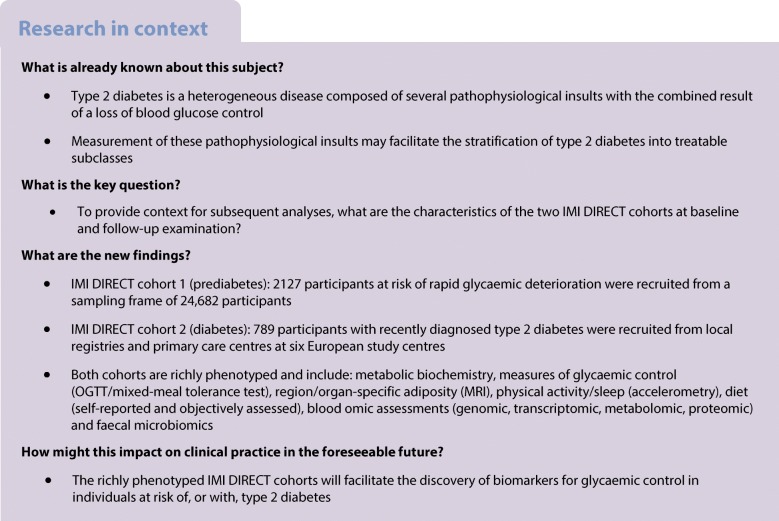



The global prevalence of type 2 diabetes is burgeoning. There is no cure, nor are there treatments effective enough to halt the progression of the disease. The burden the disease conveys at a societal and personal level is enormous, with an estimated world prevalence in 2017 of around 425 million people with type 2 diabetes and a further 352 million at risk of developing the disease [1]. The global cost of diagnosing and treating the disease and its complications in 2017 was estimated to be around €730 billion [1]. This bleak picture emphasises the profound shortcomings in our understanding of type 2 diabetes aetiology and pathogenesis, and the inadequate tools available with which to combat the disease.

Like some other complex diseases, the clinical presentation and prognosis of type 2 diabetes is heterogeneous. The risk conveyed by established diabetogenic factors such as obesity, physical inactivity and certain dietary components varies widely from one person to the next, as does the response to interventions targeting these risk factors. This is also true for those in whom diabetes is manifest, with response to glucose-lowering therapies, occurrence of adverse events and rates of progression being variable and hard to predict.

The diagnosis of type 2 diabetes is relatively straightforward, relying primarily on evidence of chronically elevated blood glucose concentrations [2]. However, elevated blood glucose concentrations can be the consequence of multiple defects in energy metabolism occurring across several organs and tissues [3–5] caused by myriad acquired or inherited factors. Thus, type 2 diabetes as currently defined characterises a collection of underlying pathologies [6], each with the common feature of elevated blood glucose that may require tailored therapies. The stratification of type 2 diabetes into treatable subclasses might be possible if accessible biomarkers of the disease’s underlying pathologies were known.

Although improving the management of type 2 diabetes through subclassification may lead to more focused treatment, susceptibility to risk factors and response to treatments also vary. Thus, stratifying patient populations into subgroups defined using biomarkers quantifying susceptibility to risk factors and responsiveness to specific therapeutics would further enhance our ability to treat and ideally prevent the disease.

The Innovative Medicines Initiative (IMI) Diabetes Research on Patient Stratification (DIRECT) Consortium is a collaboration among investigators from some of Europe’s leading academic institutions and pharmaceutical companies [7]. The overarching objective of IMI DIRECT is to discover and validate biomarkers of glycaemic deterioration before and after the onset of type 2 diabetes. To this end, we established two new multicentre prospective cohort studies comprised of adults from northern Europe at risk of or with recently diagnosed type 2 diabetes. Within these cohorts, a comprehensive array of risk factors, intermediate phenotypes and metabolic outcomes were repeatedly assessed using cutting-edge technologies. The richly phenotyped IMI DIRECT cohorts will facilitate the discovery of biomarkers for glycaemic control in individuals at risk of or with type 2 diabetes.

Here we describe the characteristics of the two IMI DIRECT cohorts at baseline and at the two major follow-up visits up to 48 months later, to provide context for those subsequently analysing and reviewing studies based on these data. We also consider these results in the context of the implemented protocols and plans outlined at the beginning of the project, as described previously [7].

## Methods

The rationale and design of the epidemiological cohorts within IMI DIRECT are reported elsewhere [7]; here we provide data and information about key variables and methods, respectively, that were not described in the rationale and design paper published previously.

Approval for the study protocol was obtained from each of the regional research ethics review boards separately and all participants provided written informed consent at enrolment. The research conformed to the ethical principles for medical research involving human participants outlined in the declaration of Helsinki.

### Recruitment, enrolment and eligibility

**Fig. 1:**
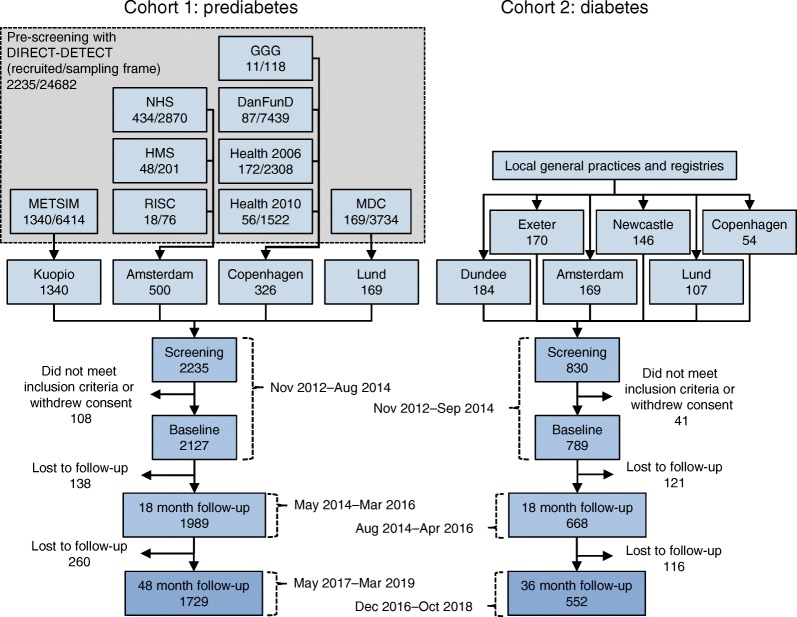
Participant flow of cohorts 1 and 2. DanFunD, Danish Functional Disability study; GGG, Gut, Grain and Greens study; HMS, Hoorn Meal Study; METSIM, Metabolic Syndrome in Men study; NHS, New Hoorn Study; RISC, Relationship between Insulin Sensitivity and Cardiovascular disease cohort

The derivations of cohort 1 and cohort 2 are shown in Fig. 1. The sampling frame for cohort 1 comprised four existing prospective cohort studies: Metabolic Syndrome in Men (METSIM, Finland) [8]; Relationship between Insulin Sensitivity and Cardiovascular disease (RISC) [9], Hoorn Meal Study (HMS) and New Hoorn Study (NHS) [10] (Netherlands); Health2010 [11], Health2006 [12], Danish Study of Functional Disorders (DanFunD) [13] and Gut, Grain and Greens (GGG) [14] studies (Denmark) and Malmö Diet and Cancer (MDC) study (Sweden) [15]. Participants for cohort 2 were identified through general practice and other registries, as described previously [7].

After excluding participants who did not meet the inclusion criteria or whose data failed quality control, a total of 2127 participants at risk of diabetes and 789 participants with type 2 diabetes were retained in cohort 1 and cohort 2, respectively. In cohort 1, emphasis was placed on recruiting participants deemed at high risk of type 2 diabetes according to ADA 2011 HbA_1c_ criteria (HbA_1c_ 40–48 mmol/mol [5.7–6.4%]) [2, 7]. As anticipated during the design phase, the sampling frame contained too few participants that fulfilled this criterion; thus, we proceeded to enrol participants with progressively lower HbA_1c_ concentrations, who were also considered at highest risk of glycaemic deterioration according to the DIRECT-DETECT risk algorithm (based on age, BMI, waist circumference, use of antihypertensive medication, smoking status and parental history of type 2 diabetes) applied to the parent cohort sampling frame [7, 16].

In cohort 1, 1989 (93%) participants enrolled at baseline also attended the first major follow-up visit at a mean of 18.6 (SD 1.4) months and 1729 (81%) attended the final follow-up visit 30.8 (SD 1.3) months later. In cohort 2, 668 (85%) participants enrolled at baseline attended their first major follow-up visit at 18.2 (SD 0.6) months and 552 (70%) attended the final follow-up visit 18.2 (SD 1.0) months later.

### Glycaemic biochemistry assays

Plasma glucose, insulin and C-peptide assays for cohort 1 were carried out centrally at the University of Eastern Finland (Kuopio, Finland), where plasma glucose was analysed using the enzymatic glucose hexokinase method and photometric measurement on Konelab 20 XT Clinical Chemistry analyser (Thermo Fisher Scientific, Vantaa, Finland). In cohort 2, plasma insulin and C-peptide were analysed using chemiluminometric immunoassay (CLIA) (Liaison Insulin [DiaSorin, Saluggia, Italy] and Liaison C-peptide [DiaSorin]). The instrument used was DiaSorin Liaison Analyser (DiaSorin Deutschland, Dietzenbach, Germany). Plasma glucose, insulin and C-peptide assessments for cohort 2 were carried out centrally at the University of Exeter (Exeter, UK). Assessments of HbA_1c_, blood lipids, alanine aminotransferase (ALT) and aspartate aminotransferase (AST) for both cohorts were carried out centrally at the University of Exeter. Glucose was measured by the enzymatic colorimetric assay GOD-PAP using Roche MODULAR P analysers (Hoffmann-La Roche, Basel, Switzerland). Insulin was measured by electrochemiluminescence using Roche E170 Analysers (Hoffmann-La Roche). C-peptide concentrations in plasma and urine were measured by electrochemiluminescence using Roche E170 Analysers (Hoffmann-La Roche). HbA_1c_ was measured by ion-exchange high-performance liquid chromatography using Tosoh G8 analysers (Tosoh Bioscience, San Francisco, CA, USA). Each biochemical assay was performed using validated standard methods. Reference samples were included in all procedures to control for inter-assay variation and laboratories regularly participated in international external quality assessment schemes. In addition, a subset of samples was assayed for C-peptide, insulin and glucose on both sites to assess inter-laboratory variation.

### Blood lipid and liver enzyme biochemistry assays

Triacylglycerol was measured by quantitative determination with glycerol blanking. HDL-cholesterol was measured directly using polyethylene glycol-modified enzymes and dextran sulphate. When cholesterol esterase and cholesterol oxidase enzymes are modified by polyethylene glycol, they show selective catalytic activity towards lipoprotein fractions, with reactivity increasing in the order LDL < VLDL ≈ chylomicrons < HDL. Total cholesterol was measured by an enzymatic, colorimetric method. LDL-cholesterol was calculated from the total cholesterol, HDL-cholesterol and triacylglycerol concentrations using the Friedewald formula: LDL = total cholesterol – HDL-cholesterol – (triacylglycerol/2.2). ALT and AST were measured by UV absorbance without pyridoxal phosphate activation. ALT, AST, cholesterol, glucose, triacylglycerol and HDL-cholesterol were measured using a Roche MODULAR P analyser (Roche Diagnostics, Indianapolis, IN, USA). Insulin and C-peptide were measured using a Roche E170 analyser (Roche Diagnostics).

### Blood glucagon-like peptide-1 assays

Plasma concentrations of glucagon-like peptide-1 (GLP-1) were determined by drawing blood samples collected at two different time points (0 and 60 min) during the 75 g frequently sampled OGTT (fsOGTT)/mixed-meal tolerance test (MMTT) (baseline samples only). P800 tubes (Becton Dickinson, Wokingham, UK) were used to provide immediate protection from intrinsic proteolysis. Quantitative determination of active GLP-1 was achieved using MSD GLP-1 active kit (product code K150JWC; Meso Scale Diagnostics, Rockville, MD, USA). Total GLP-1 was assayed using MSD GLP-1 total kit (product code K150JVC; Meso Scale Diagnostics).

### Abdominal MRI analyses

The volume of adipose tissue was measured in litres using MRI, as described elsewhere [17]. Total abdominal adipose tissue (TAAT) may be separated into intra-abdominal adipose tissue (IAAT), also known as ‘visceral’ fat, and abdominal subcutaneous adipose tissue (ASAT). IAAT is the volume of adipose tissue within the abdominal cavity. TAAT is the sum of IAAT and ASAT. Liver and pancreas fat and iron (T2*) were derived simultaneously using a multiecho MRI technique, as previously described [17, 18]. This method has the advantage over single voxel MR spectroscopy in that regional differences in ectopic fat distribution can be measured. Furthermore, it is often possible to obtain a single slice quantification of the liver and pancreas, allowing simultaneous measurement of fat and iron within two separate organs. A biexponential curve-fitting model was used to derive the relative signal contributions from fat and water from the many images normally obtained with the multiecho sequence. Briefly, tissue with no fat infiltration generates a very smooth decay curve, whereas tissue containing a higher level of fat is characterised by significant oscillations throughout the decay curve [18]. A further output from the multiecho technique is T2* tissue values; as changes in these are indicative of iron content, this provides a clinically relevant additional measurement. Tissue iron concentration (mg/g dry weight tissue) was estimated from T2* using a validated model [19].

### Diet assessment

Self-reported dietary intake was assessed by a 24 h multi-pass method, using food habit and 24 h recall questionnaires. Analysis of these diet data was undertaken using Dietplan-6 (version 6.70.43, 2013; Forestfield Software, Horsham, UK). The specific analysis methods are described in detail elsewhere [7]. We also objectively assessed diet using discriminative metabolite signatures, an approach described in detail elsewhere [20]. Briefly, each participant’s serum metabolite profile was obtained using a targeted metabolomics assay (Absolute*IDQ* p180 Kit; BIOCRATES Life Sciences, Innsbruck, Austria), which simultaneously quantifies 188 metabolites. In a previously published diet intervention study [20], serum samples had been collected in 19 participants who had undergone a metabolic ward-based supervised diet intervention. We assayed these blood samples using the BIOCRATES Absolute*IDQ* p180 Kit and derived diet-discriminatory metabolomic signatures, using previously described methods [20]. These data were then used to predict the dietary characteristics of the IMI DIRECT study participants.

### Physical activity assessment

Objective measures of physical activity were derived from triaxial accelerometers (ActiGraph GT3X+/GT3X+w/GT3X+bt; ActiGraph Co., Pensacola, FL, USA) as described previously [7]. Raw data files (.gt3x) were converted to comma separated value (.csv) format storing rawest possible accelerations for each axis at a resolution of 30 Hz using ActiLife 6 (version 6.11.5; ActiGraph Co.). All inferred measures of physical activity were calculated using PAMPRO (version uploaded 21 Oct 2015; MRC Epidemiology unit, Cambridge, UK), custom open source software available under public license (https://github.com/Thomite/pampro). Data from each axis of acceleration was auto-calibrated to local gravity. Non-wear was inferred as a vector magnitude SD of less than 4 m*g* for a consecutive period greater than 60 min. All measures presented here have been adjusted for diurnal rhythm to account for bias from non-wear removal. However, due to the wear method (non-dominant wrist fastened using the manufacturer’s non-removable hospital band), intermittent non-wear time was rare. The main physical activity estimates presented here are high-pass-filtered vector magnitude (hpfVM), which infers intensity of participants’ movement in any direction at any given time (here, averaged during wear period). Time spent in established physical activity intensities by physical activity energy expenditure was estimated using calculated hpfVM cutpoints: sedentary (<48 m*g* hpfVM), light (48–154 m*g* hpfVM), moderate (154–389 m*g* hpfVM) and vigorous (>389 m*g* hpfVM). The methods used to infer these measures have been validated and described in detail elsewhere [21].

### DNA extraction and genotyping

DNA extraction was carried out using Maxwell 16 Blood DNA purification kits and a Maxwell 16 semi-automated nucleic acid purification system (Promega, Southampton, UK). Genotyping was conducted using the Illumina HumanCore array (HCE24 v1.0) and genotypes were called using Illumina’s GenCall algorithm. Samples were excluded for any of the following reasons: call rate <97%; low or excess mean heterozygosity; sex discordance and monozygosity. Genotyping quality control was then performed to provide high-quality genotype data for downstream analyses using the following criteria: call rate <99%; deviation from Hardy–Weinberg equilibrium (exact *p* < 0.001); variants not mapped to human genome build GRCh37 and variants with duplicate chromosome positions (a total of 30,318 markers were excluded). A total of 3032 samples and 517,958 markers across the two studies passed quality control procedures. We took autosomal variants with MAF >1% that passed quality control and constructed axes of genetic variation using principal components analysis implemented in the GCTA (version 1.24.4, downloaded from https://cnsgenomics.com/software/gcta/#Download) software to identify ethnic outliers defined as non-European ancestry using the 1000 Genomes samples as reference. We identified six individuals as ethnic outliers.

### Additional measures (not presented here)

Biomarker discovery analyses using these data also employ additional measures (including ‘omic’ measures), which are outside the scope of this cohort description. Additional measures that are not described here include transcriptomics (RNA sequencing from fasting whole blood), microbiomics (DNA isolation and deep sequencing in faecal samples), proteomics (targeted array in fasting plasma) and metabolomics (targeted and untargeted assays in fasting plasma). GAD/islet antigen-2 assessments from fasting serum samples were also undertaken. Data from the Recent Physical Activity Questionnaire (RPAQ) and sleep diaries were also collected in subcohorts.

### Statistical power of study

A detailed section on sample size and power for the study is available in the previously published rationale and design paper [7]. Briefly, statistical power will vary depending on a number of factors specific to the analysis to be carried out, such as biomarker effect sizes, variance/frequency of outcome and biomarker, statistical modelling methods employed, number of tests (multiple testing adjustment) and of course sample size, available for the relevant variables included in the model. The dataset will therefore be well powered for some analyses while it may be underpowered for other analyses and will thus be covered in detail for the specific scenarios in subsequent analyses.

### Statistical methods for descriptive data

Based on ADA 2011 diagnostic criteria, impaired fasting plasma glucose is 5.6–6.9 mmol/l (100–125 mg/dl), impaired glucose tolerance is 2 h 75 g OGTT plasma glucose 7.8–11.0 mmol/l (140–199 mg/dl) and prediabetes HbA_1c_ is 40–48 mmol/mol (5.7–6.4%) [2]. Accordingly, below and above these cut-offs was considered ‘normal’ and ‘diabetic’ ranges, respectively. Cohort 1 was stratified into two categories of blood glucose level: normal glucose regulation (NGR) and impaired glucose regulation (IGR). NGR was defined as the HbA_1c_, fasting glucose and 2 h glucose values being within the normal ranges for each measure. IGR was defined as there being impaired values in at least one of HbA_1c_, fasting glucose or 2 h glucose. Cohort 2 was stratified into treatment categories: lifestyle advice only or metformin plus lifestyle advice. Descriptive statistics are presented as mean ± SD. Pairwise Pearson correlations were calculated for all key variables described here. For these analyses, continuous variables were first inverse normal transformed and then adjusted for age, sex and study centre by two-step residual regression. We present the same type of data for anthropometric and glycaemic variables for the main follow-up visits, as well as the differences for these variables between the baseline and the final follow-up visit measures (follow-upΔ = final follow-up value – baseline value). We also calculated pairwise Pearson correlations for the follow-upΔ values; for these analyses, continuous variables were first inverse normal transformed and then adjusted for age, sex, study centre and days since baseline visit by two-step residual regression. All statistics were computed using R software version 3.4.0 [22]. The IMI DIRECT data release version used for the analyses in this article was direct_03-29-2019.

### Glycaemic trait modelling

Glycaemic traits were derived from the 75 g fsOGTT (sampling at 0, 15, 30, 45, 60, 90, 120 min) and MMTT (sampling at 0, 30, 60, 90, 120 min) for cohort 1 and cohort 2, respectively. Analyses used a mathematical model that describes the relationship between insulin secretion and glucose concentration [23, 24]. The model expresses insulin secretion as the sum of two components, the first of which represents the dependence of insulin secretion on absolute glucose concentration at any time during the fsOGTT/MMTT, through a dose–response function. Characteristic parameters of the dose–response relationship are the mean slope over the observed glucose range, denoted as glucose sensitivity. The dose–response relationship is modulated by a potentiation factor, which accounts for the fact that during acute stimulation, insulin secretion is higher on the descending phase of hyperglycaemia than at the same glucose concentration on the ascending phase. In participants with NGR and normal insulin secretion, the potentiation factor typically increases from baseline to the end of a 2 h OGTT [25]. To quantify this excursion, the ratio between the 2 h and the baseline value was calculated. This ratio is denoted as potentiation ratio and reflects late insulin release. The second insulin secretion component represents the dependence of insulin secretion on the rate of change of glucose concentration. This component is termed derivative component and is determined by a single parameter, denoted as rate sensitivity. Rate sensitivity reflects early insulin release [25].

The model parameters were estimated from glucose and C-peptide concentrations by regularised least-squares, as previously described [23]. Regularisation involves the choice of smoothing factors, which were selected to obtain glucose and C-peptide model residuals with SDs close to expected measurement error (~1% for glucose and ~4% for C-peptide). Insulin secretion rates were calculated from the model every 5 min. The integral of insulin secretion during the fsOGTT was denoted as total insulin output.

The validity of the fsOGTT and MMTT for the assessment of insulin sensitivity has been shown in the original publications presenting the indices [26–28]. In the studies, the OGTT/MMTT indices are compared with values obtained by euglycaemic glucose clamp. The validity of the beta cell function model is supported by numerous studies [25]. For beta cell function, it is not possible to validate an OGTT/MMTT method against the classical tests with glucose intravenous infusion, due to the presence of the incretin effect. However, it has been shown that the estimated beta cell dose–response is consistent with the graded glucose infusion test across the spectrum of glucose tolerance [29, 30].

## Results

### Cohort 1 (prediabetes)

Of 2235 enrolled participants in cohort 1, 2127 passed all inclusion, exclusion and quality control criteria. Of these, 1419 (67%) had IGR according to at least one ADA category for HbA_1c_, fasting glucose or 2 h glucose [2] and were thus within the target ‘prediabetes’ range. A total of 693 participants (33% of cohort 1) displayed NGR according to all three glycaemic measures. Participants with prevalent type 2 diabetes (*n* = 105) or who withdrew from the study (*n* = 3) were excluded from further analyses.

The number of participants enrolled into cohort 1 varied between centres, with the Finnish subcohort being the largest (providing 58% [*n* = 1240]) of the total cohort 1 baseline sample. The other centres in the Netherlands, Denmark and Sweden enrolled 22% (*n* = 473), 13% (*n* = 275) and 7% (*n* = 139) of the total cohort, respectively.

The ratio of men to women varied in each subcohort, with all participants at the Finnish centre being male, and 43%, 45% and 29% being male in the subcohorts from the Netherlands, Denmark and Sweden, respectively.

**Fig. 2:**
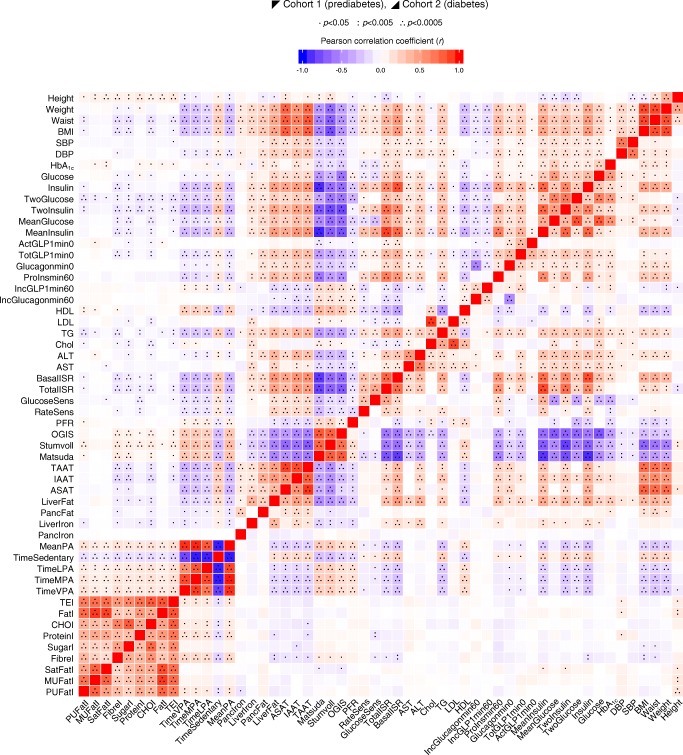
Pairwise correlation matrix. Fill colour indicates Pearson correlation coefficient (*r*), where positive is denoted by red fill, inverse by blue fill and magnitude by intensity. Cohorts 1 and 2 are separate, above and below the diagonal, respectively. All continuous variables were normally transformed and adjusted for age, sex and study centre. ActGLP1min0, fasting intact GLP-1 concentration; BasalISR, fasting insulin secretion; CHOI, carbohydrate intake; Chol, total cholesterol; DBP, diastolic blood pressure; FatI, fat intake; FibreI, fibre intake; Glucagonmin0, fasting glucagon; Glucose, fasting glucose; GlucoseSens, glucose sensitivity; HDL, fasting HDL-cholesterol; IncGLP1min60, 1 h GLP-1 increment; IncGlucagonmin60, 1 h glucagon increment; Insulin, fasting insulin; LDL, fasting LDL-cholesterol; LiverFat, liver fat; LiverIron, liver iron content; LPA, light physical activity (% of time); Matsuda, Matsuda insulin sensitivity index; MeanGlucose, mean 2 h glucose; MeanInsulin, mean 2 h insulin; MPA, moderate physical activity (% of time); MUFatI, monounsaturated fat intake; OGIS, 2 h insulin sensitivity; PA, mean physical activity intensity; hpfVM; PancFat, pancreatic fat; PancIron, pancreatic iron content; PFR, potentiation factor ratio; ProInsmin60, 1 h intact proinsulin; ProteinI, protein intake; PUFatI, polyunsaturated fat intake; RateSens, rate sensitivity; SatFatI, saturated fat intake; SBP, systolic blood pressure; SPA, sedentary (% of time); Stumvoll, Stumvoll insulin sensitivity index; SugarI, sugar intake; TEI, total energy intake; TG, fasting triacylglycerol; TotalISR, integral of total insulin secretion; TotGLP1min0, fasting total GLP-1 concentration; TwoGlucose, 2 h glucose; TwoInsulin, 2 h insulin; VPA, vigorous physical activity (% of time)

**Fig. 3:**
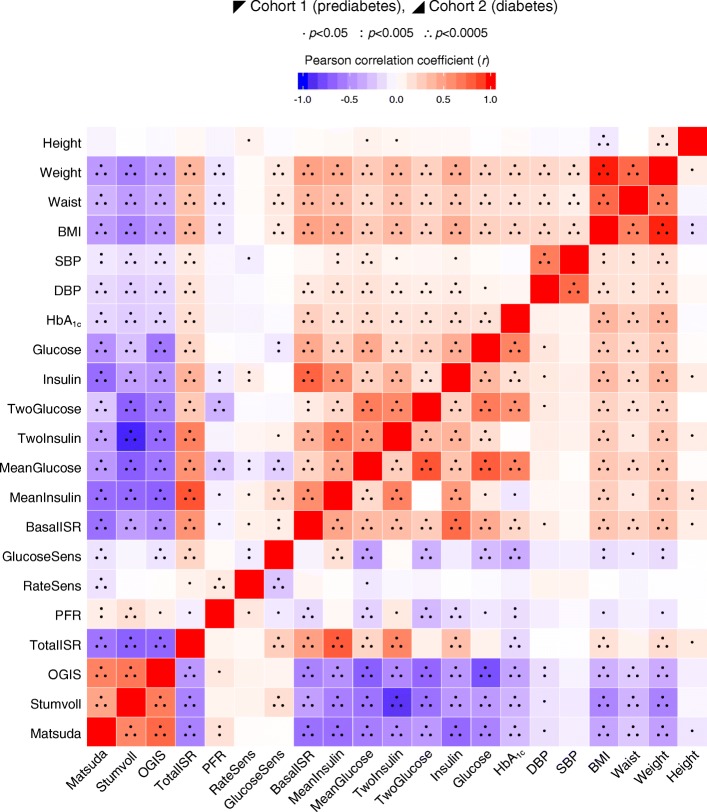
Pairwise correlation matrix of follow-up Δ (difference between 48 month follow-up assessment and baseline visit). Fill colour indicates Pearson correlation coefficient (*r*), where positive is denoted by red fill, inverse by blue fill and magnitude by intensity. Cohorts 1 and 2 are separate, above and below diagonal, respectively. All continuous variables were normally transformed and adjusted for age, sex and study centre. BasalISR, fasting insulin secretion; DBP, diastolic blood pressure; Glucose, fasting glucose; GlucoseSens, glucose sensitivity; Insulin, fasting insulin; Matsuda, Matsuda insulin sensitivity index; MeanGlucose, mean 2 h glucose; MeanInsulin, mean 2 h insulin; OGIS, insulin sensitivity (2 h OGIS); PFR, potentiation factor ratio; RateSens, rate sensitivity; SBP, systolic blood pressure; Stumvoll, Stumvoll insulin sensitivity index; TotalISR, integral of total insulin secretion; TwoGlucose, 2 h glucose; TwoInsulin, 2 h insulin.

Detailed participant baseline characteristics for cohort 1 are shown in Table 1 (and stratified by glycaemic category in electronic supplementary material [ESM] Table 1). Figure 2 shows the pairwise correlations between a selection of key phenotypic variables at baseline. Participant characteristics at the follow-up visits and the difference (Δ) between baseline and final follow-up for cohort 1 are shown in Table 2. The pairwise correlations between the baseline to final follow-up difference for anthropometric and glucose-control variables are shown in Fig. 3.

**Table 1:** Baseline clinical and phenotypic characteristics of cohorts 1 and 2

Characteristic	Cohort 1 (prediabetes)		Cohort 2 (diabetes)	
	Value	*n*	Value	*n*
Male sex, %	76	2127	58	789
Time since screening visit, months	6.4 (4.8)	2127	0.9 (0.9)	787
Age (years)	62 (6.2)	2127	62 (8.1)	787
Height, cm	174 (8)	2127	171 (9.8)	787
Weight (kg)	85 (13)	2127	89 (17)	787
Waist circumference, cm	99 (11)	2127	103 (13)	781
BMI, kg/m^2^	27.9 (4.0)	2127	30.5 (5.0)	787
Systolic blood pressure, mmHg	131 (15)	2107	131 (16)	664
Diastolic blood pressure, mmHg	81 (9.0)	2107	75 (9.5)	664
HbA_1c_, mmol/mol	37 (2.9)	2113	46 (5.8)	784
HbA_1c_, %	5.5 (0.3)	2113	6.4 (0.5)	784
Fasting glucose, mmol/l	5.7 (0.6)	2126	7.2 (1.4)	787
Fasting insulin, pmol/l	78.2 (54.5)	2124	106.6 (70.9)	787
Fasting HDL-cholesterol, mmol/l	1.3 (0.4)	2123	1.2 (0.4)	789
Fasting LDL-cholesterol, mmol/l	3.2 (0.9)	2123	2.3 (1.0)	781
Fasting triacylglycerol, mmol/l	1.4 (0.6)	2123	1.5 (0.9)	789
ALT, U/l	18 (12)	2120	26 (14)	789
AST, U/l	27 (10)	2052	26 (12)	789
Total cholesterol, mmol/l	5.1 (1)	2123	4.2 (1.2)	789
Fasting intact GLP-1 concentration, pg/ml	0.41 (0.59)	2121	0.67 (1.05)	782
Fasting total GLP-1 concentration, pg/ml	6.5 (4.4)	2120	9.4 (9)	780
Fasting glucagon, pg/ml	98 (41)	2116	111 (51)	758
1 h intact proinsulin, pg/ml	19 (11.7)	578	21 (13.6)	382
1 h GLP-1 increment, pg/ml	9.3 (12.1)	2103	9.8 (12.5)	774
1 h glucagon increment, pg/ml	−10.7 (38)	2097	−3.9 (51)	746
Mean 2 h glucose, mmol/l	7.7 (1.5)	2126	9.3 (2)	779
Mean 2 h insulin, pmol/l	383 (260)	2126	457 (275)	779
2 h glucose, mmol/l	5.9 (1.6)	2127	8.6 (2.8)	786
2 h insulin, pmol/l	48 (48)	2102	445 (348)	786
Fasting insulin secretion, pmol min^−1^ m^−2^	106 (40)	2126	137 (48)	779
Integral of total insulin secretion, nmol/m^2^	52 (18)	2126	44 (14)	779
Glucose sensitivity, pmol min^−1^ m^−2^ (mmol/l)^−1^	113 (55)	2126	83 (55)	779
Rate sensitivity, pmol m^−2^ (mmol/l)^−1^	921 (699)	2126	1124 (1082)	779
Potentiation factor ratio, dimensionless	1.7 (0.6)	2126	1.4 (0.6)	777
Insulin sensitivity (2 h OGIS), ml min^−1^ m^−2^	381 (59)	2118	298 (69)	775
Stumvoll insulin sensitivity index, ml min^−1^ kg^−1^	7.8 (2.4)	2099	5.5 (2.7)	775
Matsuda insulin sensitivity index, arbitrary units	5 (3.1)	2126	2.9 (2.2)	779
IAAT, l	5.5 (2.4)	956	5.7 (2.2)	374
ASAT, l	6.1 (2.6)	953	8.1 (3.8)	374
TAAT, l	12 (3.9)	953	14 (4.8)	374
Liver fat, %	5 (4.7)	959	8.7 (7.1)	498
Pancreatic fat, %	13 (8.9)	929	11 (7.3)	446
Liver iron content, mg/g tissue	1.3 (0.26)	958	1.4 (0.31)	498
Pancreatic iron content, mg/g tissue	1.3 (0.43)	927	1.2 (0.33)	447
Mean physical activity intensity: hpfVM, m*g*	37 (10.2)	1714	34 (9.9)	722
Sedentary, % of time	82 (4.2)	1714	83 (4.3)	722
Light physical activity, % of time	10.9 (2.3)	1714	10.4 (2.3)	722
Moderate physical activity, % of time	5.3 (1.5)	1714	4.9 (1.6)	722
Vigorous physical activity, % of time	1.5 (0.7)	1714	1.3 (0.7)	722
Total energy intake, kJ/day	8213 (3142)	2064	7699 (2519)	707
Carbohydrate intake, g/day	223 (96)	2064	213 (78)	707
Fat intake, g/day	79 (39)	2064	72 (33)	707
Protein intake, g/day	99 (44)	2064	87 (31)	707
Sugar intake, g/day	96 (53)	2064	85 (43)	707
Fibre intake, g/day	20 (9.8)	2064	19 (8.4)	707
Saturated fat intake, g/day	29 (16)	2064	26 (14)	707
Monounsaturated fat intake, g/day	27 (17)	2064	24 (13)	707
Polyunsaturated fat intake, g/day	13 (8.2)	2064	12 (8)	707

Data are mean (SD) except for sex, which is *n*%, and reflect the data available at the time of publication

Values are untransformed and unadjusted

**Table 2:** Follow-up and follow-up Δ in clinical and phenotypic characteristics of cohorts 1 and 2

Characteristic	Cohort 1 (prediabetes)					Cohort 2 (diabetes)				
	18 Months		48 Months		Baseline to 48 months Δ	18 Months		36 Months		Baseline to 36 months Δ
	Value	*n*	Value	*n*	Value	Value	*n*	Value	*n*	Value
Male sex, %	77	1989	79	1729		60	668	61	552	
Time since previous visit, months	18.6 (1.4)	1989	30.8 (1.3)	1729		18.2 (0.6)	668	18.1 (1.0)	552	
Age, years	63.6 (6.1)	1989	65.9 (6.1)	1729		63.8 (7.7)	668	65.4 (7.7)	552	
Height, cm	174 (7.9)	1983	175 (7.9)	1729	−0.39 (1.01)	171 (9.8)	662	171 (9.9)	551	−0.5 (1.29)
Weight, kg	85 (13.5)	1981	85.3 (13.7)	1729	−0.16 (4.6)	89.6 (16.9)	664	89.3 (16.6)	548	−0.13 (5.5)
Waist circumference, cm	99 (11)	1981	100 (11)	1728	0.8 (5.9)	103 (13)	661	104 (13)	546	1.8 (6.4)
BMI, kg/m^2^	27.9 (4)	1980	28 (4.1)	1729	0.07 (1.6)	30.5 (5)	661	30.5 (4.9)	548	0.13 (1.9)
Systolic blood pressure, mmHg	131 (16)	1983	132 (16)	1728	1.7 (14)	130 (16)	667	132 (16)	550	0.6 (15)
Diastolic blood pressure, mmHg	80 (8.8)	1983	81 (9.7)	1728	0.1 (8.2)	74 (9.3)	665	74 (9.6)	548	−1.2 (8.9)
HbA_1c_, mmol/mol	37.9 (3.1)	1980	40.3 (3.4)	1668	3.2 (2.3)	48.5 (8.9)	662	48.1 (9.9)	545	1.7 (8.8)
HbA_1c_, %	5.6 (0.3)	1980	5.8 (0.3)	1668	0.3 (0.2)	6.6 (0.8)	662	6.6 (0.9)	545	0.2 (0.8)
Fasting glucose, mmol/l	5.8 (0.6)	1977	6.0 (0.6)	1679	0.3 (0.5)	7.8 (1.8)	658	7.9 (2)	509	0.8 (1.9)
Fasting insulin, pmol/l	84.7 (61.7)	1975	85.4 (59.6)	1713	8.6 (42.3)	118.8 (78.6)	655	119.3 (75.4)	496	12 (62.9)
2 h glucose, mmol/l	6.1 (1.7)	1975	6.5 (2)	1673	0.6 (1.7)	9.5 (3.3)	659	9.9 (3.4)	505	1.3 (3.2)
2 h insulin, pmol/l	55.3 (53.7)	1950	59 (57.6)	1687	11.2 (43.5)	449.7 (313.4)	648	445.7 (325.2)	488	10.4 (289.8)
Mean 2 h glucose, mmol/l	8 (2)	1974	8 (2)	1054	0.4 (1.3)	10 (3)	650	10 (3)	494	1.1 (2.3)
Mean 2 h insulin, pmol/l	425 (280)	1974	437 (287)	1054	37.2 (190)	476 (278)	650	466 (283)	494	6.7 (207)
Fasting insulin secretion, pmol min^−1^ m^−2^	103 (41)	1974	113 (44)	1054	5.7 (29)	145 (52)	650	148 (54)	494	10.7 (41)
Glucose sensitivity, pmol min^−1^ m^−2^ mmol/l^−1^	109 (55)	1974	114 (55)	1054	2.3 (54.3)	81 (57)	650	77 (62)	494	−6.3 (53.1)
Rate sensitivity, pmol m^−2^ mmol/l^−1^	833 (673)	1974	859 (627)	1054	−65 (627)	1176 (1290)	650	1011 (871)	494	−56 (1181)
Potentiation factor ratio, dimensionless	2 (1)	1973	2 (1)	1052	0.1 (0.8)	1 (1)	650	1 (1)	491	0 (0.7)
Integral of total insulin secretion, nmol/m^2^	50 (19)	1974	54 (18)	1054	1.5 (12.6)	45 (14)	650	45 (14)	494	1 (11.4)
Insulin sensitivity (2 h OGIS), ml min^−1^ m^−2^	369 (60.8)	1963	353 (58.6)	1046	−21 (46.1)	277 (53.9)	641	276 (53.9)	476	−23.2 (71)
Stumvoll insulin sensitivity index, ml min^−1^ kg^−1^	7.6 (2.6)	1941	7.4 (2.7)	1041	−0.4 (1.8)	5.3 (2.7)	640	5.2 (2.7)	474	−0.4 (2.1)
Matsuda insulin sensitivity index, arbitrary units	4.7 (3.1)	1974	4.4 (2.8)	1054	−0.6 (2.2)	2.4 (1.7)	649	2.4 (1.6)	491	−0.5 (1.9)

Descriptive statistics shown are mean (SD) except for sex, which is *n*%

Values are untransformed and unadjusted. Follow-up Δ is mean (SD) of difference between characteristic value between follow-up assessment and baseline visits

Briefly, at baseline, participants had a mean (SD) age of 62 (6.2) years, BMI 27.9 (4.0) kg/m^2^, HbA_1c_ 37 (2.9) mmol/mol [5.5 (0.27)%], fasting glucose 5.7 (0.6) mmol/l, 2 h glucose 5.9 (1.6) mmol/l, fasting insulin 10.9 (7.6) pmol/l, glucose sensitivity 113 (55) pmol min^−1^ m^−2^ (mmol/l)^−1^ and insulin sensitivity (2 h oral glucose insulin sensitivity [OGIS]) 381 (59) ml min^−1^ m^−2^. Participants had a 0–48 month mean (SD) difference in fasting plasma glucose levels of 0.3 (0.2) mmol/l.

### Cohort 2 (diabetes)

Of 830 individuals in cohort 2 enrolled to attend the screening visit, 789 passed all inclusion, exclusion and quality control criteria. Of these, 272 were treated with lifestyle modification plus metformin and 517 were treated with lifestyle intervention only. Participants who withdrew consent, who were receiving any other oral hypoglycaemic agent or who reported ever receiving insulin treatment were excluded (*n* = 41).

Of the participants in cohort 2 at baseline, the UK (Dundee, Exeter, Newcastle), Dutch (Amsterdam), Swedish (Lund) and Danish (Copenhagen) study centres enrolled 21% (*n* = 167), 18% (*n* = 141), 21% (*n* = 166), 21% (*n* = 167), 12% (*n* = 96) and 7% (*n* = 52) of the total cohort, respectively; 52–63% of the subcohort participants were male.

Detailed participant characteristics and key variables for cohort 2 at baseline are shown in Table 1 (and stratified by treatment category in ESM Table 1). Figure 2 shows the pairwise correlation matrix for key variables at baseline adjusted for age, sex and study centre. Participant characteristics for follow-up visits and the difference between the baseline and final follow-up visit (Δ) for cohort 2 are shown in Table 2. A pairwise correlation matrix for the anthropometric and glucose-control Δ variables are shown in Fig. 3.

Briefly, at baseline, participants had a mean (SD) age 62 (8.1) years, BMI 30.5 (5.0) kg/m^2^, HbA_1c_ 46.5 (5.8) mmol/mol [6.4 (0.53)%], fasting glucose 7.2 (1.4) mmol/l, 2 h glucose 8.6 (2.8) mmol/l, fasting insulin 107 (71) pmol/l, glucose sensitivity 83 (55) pmol min^−1^ m^−2^ (mmol/l)^−1^ and insulin sensitivity (2 h OGIS) 298 (69) ml min^−1^ m^−2^. Participants had a 0 months to 18 months mean difference in fasting plasma glucose levels of 0.8 (1.9) mmol/l.

### Genetic population substructure

**Fig. 4:**
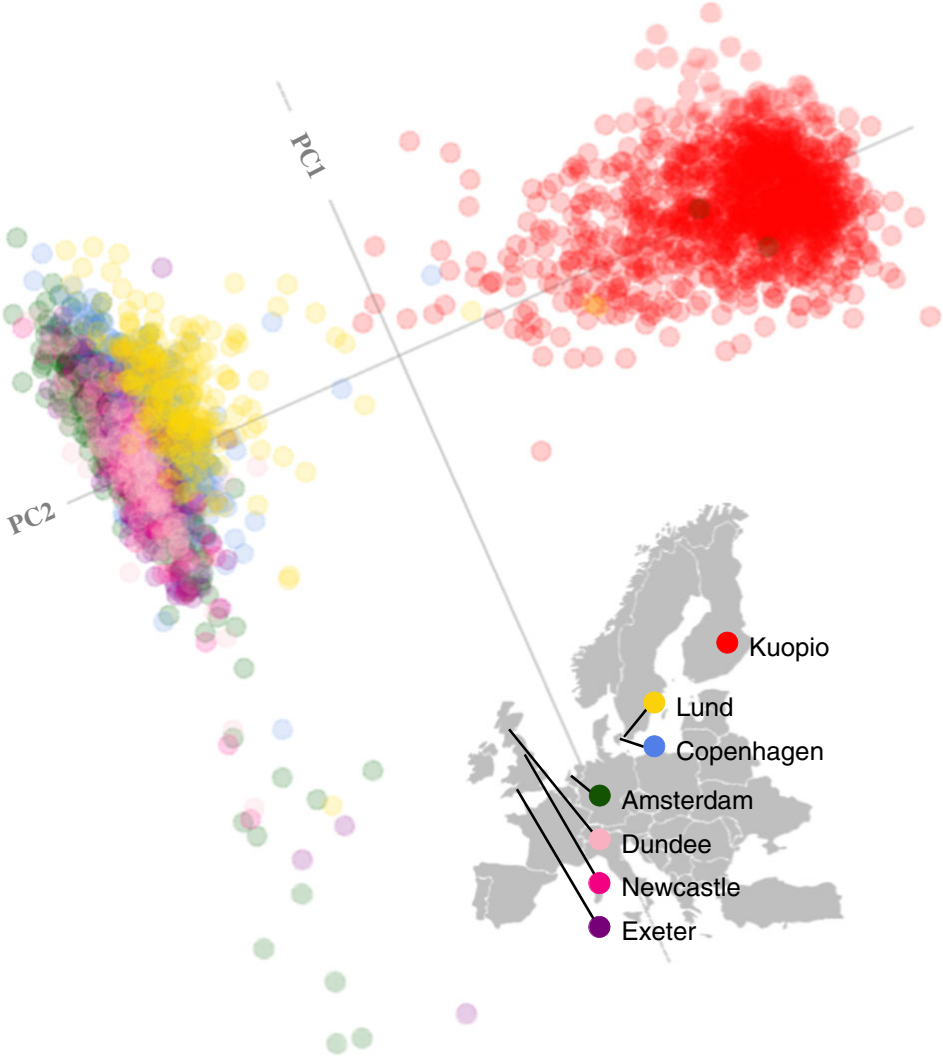
Genetic population structure within the cohorts. A statistical summary of genetic data from cohorts 1 and 2 (combined) based on principal component axis one (PC1) and axis two (PC2). Points are coloured according to recruitment centre

As some study centres enrolled participants into both cohorts, we elected to characterise the genetic population substructure across the cohorts by study centre (i.e. pooling both cohorts at a given centre where possible). Genetic substructures closely map to the geographic location of the populations [31], indicating ethnic homogeneity within regions from which the cohorts were recruited, whereas there is far greater heterogeneity between centres, the latter driven mainly by the inclusion of Finnish participants. This is illustrated in Fig. 4 where Finnish participants form a distinct cluster (to the north east) compared with the population from the other cohorts.

## Discussion

Here, we report the characteristics of the IMI DIRECT cohorts at baseline, at 18 months follow-up, and at 48 or 36 months follow-up (for cohorts 1 and 2 respectively) for glycaemic deterioration and consider these results in the context of the implemented protocols and the plans outlined in the design and rationale paper published previously [7]. The descriptive statistics, pairwise correlations and genetic substructures presented in this article are not intended for aetiological inference; instead, the purpose is to provide context and details for subsequent IMI DIRECT papers, as well as to inform scientists outside the Consortium who might in the future consider using the IMI DIRECT data in their research.

Major advances in technologies and methods over the past decade make high-resolution quantification of disease phenotypes and processes possible in large sample collections. Applying modern assays to historical biosamples is particularly useful when studying processes that take decades to unfold. However, biosamples often degrade during long-term storage and many older studies did not deploy the advanced phenotyping methods available today. Recognising these limitations, we designed and initiated two state-of-the-art prospective cohort studies as part of the IMI DIRECT Consortium. Designed for biomarker discovery in glycaemic deterioration and diabetes progression, the IMI DIRECT cohorts include conventional and cutting-edge phenotyping techniques and technologies that are repeated on multiple occasions during a follow-up period of up to 48 months (currently ongoing). We note that the subsequent biomarker discovery analyses using these cohorts will combine the clinical phenotypic data described in this paper with omic measures such as transcriptomics (RNA sequencing from fasting whole blood), microbiomics (DNA isolation and deep sequencing in faecal samples), proteomics (targeted array in fasting plasma) and metabolomics (targeted and untargeted assays in fasting plasma).

The recruitment strategies for the two IMI DIRECT cohorts differed in that cohort 1 focused on recruiting participants from an existing large sample frame (*N* = 24,682) derived from established prospective cohort studies, whereas cohort 2 used clinical registries to identify eligible participants. The strategy for recruiting participants from existing prospective studies for cohort 1 facilitated access to data that were used to predict risk of rapid glycaemic deterioration. However, despite the relatively large sampling frame, it was necessary to enrol lower-risk participants in order to achieve the target sample size; in doing so, we recognised that this would likely reduce the overall rate of glycaemic deterioration in the cohort, although the generalisability of the study’s findings will be greater. In cohort 2, we fell slightly short of the target sample size of 1000 participants (*N* = 789 with complete and high-quality data eventually enrolled). This reflects the difficulties in engaging some general practices, which was necessary to access diabetes registries in some regions.

We stratified cohorts 1 and 2 by broad glucose-control category (overtly normoglycaemic or impaired glycaemic regulation in any ADA category) or treatment category (lifestyle only vs lifestyle plus metformin), respectively, to reflect the basic stages of progression at baseline for descriptive purposes.

The two IMI DIRECT cohorts are not identical. However, they share many methodology parallels that permit complimentary analyses to be performed, such as determining whether biomarkers for glycaemic deterioration are conditional on disease state. Nevertheless, several key differences in the protocols (e.g. fsOGTT vs MMTT) should be considered when interpreting results. We also note a difference in missing accelerometry (physical activity) data between cohort 1 (19%) and cohort 2 (8.5%); we were unable to definitively explain this discrepancy. Partitioning change from error is very challenging when variables are assessed at only two time points owing to regression to the mean. Notwithstanding this, we note a modest 0 month to 48 month difference in mean (SD) fasting plasma glucose levels, 0.3 (0.5) mmol/l and 0.8 (1.9) mmol/l in cohort 1 and cohort 2, respectively (Table 2), which likely reflects the relatively brief between-visit interval. Furthermore, we note that the 0.5 mmol/l and 1.9 mmol/l SDs in these differences, for cohort 1 and cohort 2, respectively, suggest the potential heterogeneity in changes in glycaemic control within each cohort. With this in mind, the IMI DIRECT cohorts are being followed further with record-linkage through 2026. It should also be noted that the IMI DIRECT cohorts are predominantly of European ancestry; therefore results from subsequent analyses on these cohorts will need to be replicated in other cohorts of relevant ancestry before generalising findings to other ethnicities. Finally, we note that the results presented here reflect the data available at publication and as long-term follow-up progresses additional data will accrue.

## Conclusion

The study described here is being used to unravel the heterogeneous nature of glycaemic deterioration in individuals at risk of diabetes and in those with diabetes, and to discover biomarkers that might prove useful for patient stratification and therapeutic optimisation. As more prospective data are accrued, the IMI DIRECT cohorts will grow in value. In the long term, the IMI DIRECT Consortium intends to make these data available to other researchers through a managed-access repository.

## Electronic supplementary material

ESM Table (PDF 124 kb)

## Data Availability

Requests for access to IMI DIRECT data, including data presented here, can made to DIRECTdataaccess@Dundee.ac.uk.

## Abbreviations

ALT: Alanine aminotransferase
ASAT: Abdominal subcutaneous adipose tissue
AST: Aspartate aminotransferase
DIRECT: Diabetes Research on Patient Stratification
fsOGTT: Frequently sampled OGTT
GLP-1: Glucagon-like peptide-1
hpfVM: High-pass-filtered vector magnitude
IAAT: Intra-abdominal adipose tissue
IGR: Impaired glucose regulation
IMI: Innovative Medicines Initiative
MMTT: Mixed-meal tolerance test
NGR: Normal glucose regulation
OGIS: Oral glucose insulin sensitivity
TAAT: Total abdominal adipose tissue
