# Supplementary material for: Discovery of biomarkers for glycaemic deterioration before and after the onset of type 2 diabetes: descriptive characteristics of the epidemiological studies within the IMI DIRECT Consortium
Source: Diabetologia. 2019 Jun 15;62(9):1601–15. doi: 10.1007/s00125-019-4906-1 (PMC6677872; doi:10.1007/s00125-019-4906-1)
Supplement: Supplementary file 1 — (PDF 124 kb) [file 125_2019_4906_MOESM1_ESM.pdf]

ESM Table 1.

		Cohort 1 (Prediabetes)			Cohort 2 (Diabetes)		
		NGR Mean (SD)	IGR Mean (SD)	NGR/IGR N	LS Mean (SD)	LS+MET Mean (SD)	NGR/IGR N
Anthropometrics	Male, %	75%	76%	693, 1419	58%	56%	508, 271
	Months since previous visit	6.8(4.9)	6.2(4.8)	693, 1419	0.8(1)	0.9(0.8)	507, 271
	Age, yrs	62(6.2)	62(6.2)	693, 1419	62(7.9)	61(8.3)	507, 271
	Height, cm	174(7.9)	174(8.1)	693, 1419	171(9.5)	171(10.2)	507, 271
	Weight, kg	83(12)	86(14)	693, 1419	89(17)	89(17)	507, 271
	Waist circumference, cm	97(10)	101(11)	693, 1419	103(14)	104(13)	502, 270
	BMI, $kg \cdot m^{-2}$	27.2(3.7)	28.3(4.1)	693, 1419	30.7(5.1)	30.3(4.8)	507, 271
	Systolic blood pressure, mmHg	129(15)	132(15)	685, 1407	132(16)	129(14)	450, 211
	Diastolic blood pressure, mmHg	79(8.7)	81(9)	685, 1407	75(9.6)	75(9.3)	450, 211
Glycaemic Control	HbA1c, mmol/mol	35(2.2)	38(2.9)	693, 1419	46(5.7)	47(5.8)	508, 266
	HbA1c, %	5.4(0.2)	5.6(0.3)	693, 1419	6.4(0.5)	6.5(0.5)	508, 266
	Fasting glucose, mmol/l	5.2(0.4)	5.9(0.5)	693, 1419	7(1.4)	7.5(1.5)	506, 271
	Fasting insulin, pmol/l	60.3(36.3)	86.8(60.3)	693, 1417	105.8(70.6)	108.1(72)	506, 271
	Fasting HDL cholesterol, mmol/l	1.4(0.3)	1.3(0.4)	690, 1418	1.2(0.4)	1.2(0.4)	508, 271
	Fasting LDL cholesterol, mmol/l	3.2(0.9)	3.2(0.9)	690, 1418	2.3(1)	2.3(0.9)	506, 266
	Fasting triglycerides, mmol/l	1.2(0.5)	1.4(0.7)	690, 1418	1.5(0.8)	1.7(0.9)	508, 271
	Alanine aminotransferase, IU/l	16(10)	19(13)	690, 1415	25(12)	28(16)	508, 271
	Aspartate transaminase, IU/l	26(9)	28(11)	669, 1369	25(10)	27(14)	508, 271
	Total cholesterol, mmol/l	5.1(1)	5.1(1)	690, 1418	4.2(1.2)	4.3(1.1)	508, 271
	Fasting intact GLP-1 concentration (pg/ml)	0.37(0.45)	0.42(0.65)	691, 1415	0.61(1.04)	0.77(1.06)	505, 267
	Fasting total GLP-1 concentration, pg /ml	6.1(4.1)	6.7(4.5)	690, 1415	9(7.6)	10.1(11.2)	502, 268
	Fasting glucagon, pg /ml	96(33)	100(44)	690, 1411	112(56)	109(38)	488, 261
	1h intact proinsulin, pg/ml	15(7.4)	20(12.7)	145, 430	22(14)	20(12.9)	251, 128
	1h GLP-1 increment, pg/ml	9.4(11.4)	9.3(12.4)	687, 1401	9.9(14)	9.4(8.9)	497, 267
	1h glucagon increment, pg/ml	-9.5(52)	-11.3(29)	684, 1398	-4.8(58)	-1.9(37)	479, 259
	Mean 2-hr glucose, mmol/ml	6.8(1.1)	8.2(1.4)	692, 1419	8.9(1.9)	10.1(2.1)	503, 267
	Mean 2-hr insulin, pmol/ml	314(203)	418(279)	692, 1419	469(289)	433(244)	503, 267
	2-hr glucose, mmol/ml	5.3(1.2)	6.2(1.8)	693, 1419	8.2(2.7)	9.5(2.9)	505, 271
	2-hr insulin, pmol/ml	35(31)	55(53)	682, 1405	455(347)	428(352)	505, 271
	Fasting insulin secretion, pmol min^{-1} m^{-2}	90(30)	114(42)	692, 1419	134(48)	141(48)	503, 267
	Integral of total insulin secretion, $nmol/m^2$	47(15)	55(18)	692, 1419	44(15)	44(13)	503, 267
	Glucose sensitivity, pmol min^{-1} m^{-2} $(mmol/l)^{-1}$	124(62)	107(51)	692, 1419	90(57)	72(50)	503, 267
	Rate sensitivity, pmol m^{-2} $(mmol/l)^{-1}$	1003(768)	880(658)	692, 1419	1122(1119)	1130(1018)	503, 267
	Potentiation factor ratio, dimensionless,	1.8(0.6)	1.7(0.6)	692, 1419	1.5(0.7)	1.3(0.4)	501, 267
	Insulin sensitivity (2-h OGIS), ml min^{-1} m^{-2}	417(49)	362(56)	689, 1414	303(74)	288(60)	500, 266
	Stumvoll insulin sensitivity index, ml min^{-1} kg^{-1}	8.7(1.7)	7.4(2.6)	681, 1403	5.6(2.8)	5.4(2.6)	500, 266
	Matsuda insulin sensitivity index, arbitrary units	6.5(3.6)	4.3(2.5)	692, 1419	3(2.3)	2.7(1.9)	503, 267
Magnetic Resonance Imaging	Intrabdominal Adipose Tissue, l	5.1(2.3)	5.6(2.4)	274, 674	6(2.2)	5.2(2.1)	247, 122
	Abdominal Subcutaneous Adipose Tissue, l	5.5(2.3)	6.3(2.7)	273, 672	8.1(3.7)	7.9(3.9)	247, 122
	Total Abdominal Adipose Tissue, l	11(3.6)	12(4)	273, 672	14(4.7)	13(4.9)	247, 122
	Liver Fat, %	3.8(3.6)	5.5(5)	276, 675	8.2(6.6)	9.9(8)	340, 153
	Pancreatic Fat, %	14(8.8)	13(9)	262, 660	11(6.7)	11(8.2)	307, 136
	Liver Iron content, mg/g tissue	1.3(0.31)	1.3(0.24)	276, 674	1.4(0.28)	1.4(0.35)	340, 153
	Pancreatic Iron content, mg/g tissue	1.3(0.63)	1.3(0.31)	262, 658	1.2(0.33)	1.2(0.31)	306, 138
Physical Activity	Average physical activity intensity – hpfVM, m*g*s	38(10.5)	37(10)	561, 1140	35(10.1)	33(9.4)	465, 248
	Sedentary physical activity, % of time	82(4.4)	82(4.1)	561, 1140	83(4.4)	84(4.1)	465, 248
	Light physical activity, % of time	10.9(2.4)	10.8(2.2)	561, 1140	10.6(2.4)	10.1(2.2)	465, 248
	Moderate physical activity, % of time	5.3(1.6)	5.2(1.5)	561, 1140	5(1.6)	4.7(1.5)	465, 248
	Vigorous physical activity, % of time	1.6(0.8)	1.5(0.7)	561, 1140	1.4(0.7)	1.2(0.6)	465, 248
Diet	Total energy intake, kJ/day	8259(3025)	8209(3201)	670, 1380	7636(2510)	7803(2548)	474, 224
	Carbohydrate intake, g/day	227(95)	221(97)	670, 1380	212(80)	215(73)	474, 224
	Fat intake, g/day	79(38)	79(39)	670, 1380	71(32)	74(35)	474, 224
	Protein intake, g/day	98(43)	99(45)	670, 1380	86(29)	89(35)	474, 224
	Sugar intake, g/day	100(53)	95(54)	670, 1380	84(42)	87(45)	474, 224
	Fibre intake, g/day	21(10.1)	20(9.6)	670, 1380	20(8.9)	18(7.2)	474, 224
	Saturated fat intake, g/day	29(16)	30(17)	670, 1380	25(14)	28(14)	474, 224
	Monunsaturated fat intake, g/day	28(18)	27(16)	670, 1380	24(12)	25(13)	474, 224
	Polyunsaturated fat intake, g/day	13(8.6)	13(8.1)	670, 1380	12(7.6)	12(8.7)	474, 224

ESM Table 1. Baseline clinical and phenotypic characteristics of Cohorts 1 and 2 by glycaemic control and treatment status. IGR: Impaired glucose regulation, NGR: Normal glucose regulation, LS: Lifestyle Treatment, LS+MET: Lifestyle and metformin treatment. Descriptive statistics shown are N, % or mean (standard deviation) as indicated. Values are untransformed and unadjusted. Data presented reflects the data available at the time of publication.
